# Screening for Missed Opportunities for Diagnosis in the ED Using eTriggers and Large Language Models

**DOI:** 10.1001/jamanetworkopen.2026.20939

**Published:** 2026-06-29

**Authors:** Clifford M. Marks, Sean Gibney, Bryan Stenson, Deesha Sarma, Cynthia Gaudet, Haadi Mombini, Thomas A. Buckley, Mario Keko, Larry A. Nathanson, Laura G. Burke, Nathan I. Shapiro, Jonathan L. Burstein, Shamai A. Grossman, Anika Parab, Alexander T. Janke, Arjun K. Manrai, Richard A. Taylor, Carlo L. Rosen, Adam Rodman, Adrian D. Haimovich

**Affiliations:** 1 Department of Emergency Medicine, Georgetown University, Washington, DC; 2 Department of Emergency Medicine, Beth Israel Deaconess Medical Center, Harvard Medical School, Boston, Massachusetts; 3 Department of Emergency Medicine, Boston Medical Center, Boston University Chobanian & Avedisian School of Medicine, Boston, Massachusetts; 4 Beth Israel Lahey Health Technology and Innovation, Beth Israel Lahey Health, Boston, Massachusetts; 5 Department of Biomedical Informatics, Harvard Medical School, Boston, Massachusetts; 6 Department of Emergency Medicine, Warren Alpert Medical School of Brown University, Providence, Rhode Island; 7 Department of Emergency Medicine, Lahey Hospital & Medical Center, UMass Chan-Lahey School of Medicine, Burlington, Massachusetts; 8 Department of Emergency Medicine, University of Michigan Medical School, Ann Arbor; 9 Department of Emergency Medicine, University of Virginia School of Medicine, Charlottesville; 10 Department of Medicine, Division of General Medicine, Beth Israel Deaconess Medical Center, Harvard Medical School, Boston, Massachusetts

## Abstract

**Importance:**

Emergency department (ED) quality review often uses administrative electronic triggers (eTriggers), but yields on detecting missed opportunities for diagnosis (MODs) are low. A commercial large language model (LLM) may help screen for MODs, yet evaluation data in real-world cohorts remain limited.

**Objective:**

To evaluate LLMs for identifying MODs in ED eTrigger cohorts.

**Design, Setting, and Participants:**

This retrospective diagnostic study of 2 eTrigger cohorts, ED discharge with return hospital admission within 72 hours and ED admission to the floor with intensive care unit (ICU) escalation within 24 hours, was conducted from April 2015 through March 2025 across 9 EDs (2 academic and 7 community) in 1 US health system. Samples included 200 encounters from the 72-hour return cohort and 100 encounters from the floor-to-ICU cohort; each case was adjudicated by 2 emergency physicians using a review process based on the Safer Dx framework.

**Exposures:**

Cases were evaluated by Claude Sonnet 4, Claude Sonnet 4.6, Claude Opus 4.6, Gemini 3 Pro, GPT-5, and GPT-5 mini.

**Main Outcomes and Measures:**

Main outcomes were sensitivity, specificity, positive predictive value, negative predictive value, area under the receiver operating characteristic curve (AUC), and reviewer-reviewer and reviewer-model concordance.

**Results:**

Among 300 sampled encounters, 12 were excluded, leaving 288 analyzed encounters (median [IQR] age, 69 [54-79] years; 135 female [46.9%]) with 39 MODs (13.5%), including 21 of 191 (11.0%) in the 72-hour return cohort and 18 of 97 (18.6%) in the floor-to-ICU cohort. Interrater agreement was 81.9% (95% CI, 77.4%-86.1%), with Gwet AC1 of 0.77 (95% CI, 0.70-0.83). In the 72-hour return cohort, model sensitivity ranged from 42.9% (95% CI, 24.5%-63.5%) for GPT-5 mini to 85.7% (95% CI, 65.4%-95.0%) for Claude Sonnet 4, specificity from 55.9% (95% CI, 48.4%-63.1%) for Claude Sonnet 4 to 82.9% (95% CI, 76.6%-87.9%) for GPT-5 mini, and AUC from 0.65 (95% CI, 0.53-0.77) for GPT-5 mini to 0.73 (95% CI, 0.61-0.85) for Claude Sonnet 4. In the floor-to-ICU cohort, sensitivity ranged from 5.6% (95% CI, 1.0-25.8%) for GPT-5 mini to 55.6% (95% CI, 33.7%-75.4%) for Claude Sonnet 4, specificity from 64.6% (53.6%-74.2%) for Claude Sonnet 4 to 97.5% (95% CI, 91.2%-99.3%) for GPT-5 mini, and AUC from 0.57 (95% CI, 0.46-0.67) for GPT-5 mini to 0.82 (95% CI, 0.73-0.91) for GPT-5. Across cohorts, LLMs showed similar discrimination but different sensitivity-specificity tradeoffs; Claude Sonnet 4 generally favored higher sensitivity, whereas GPT-5 mini favored higher specificity.

**Conclusions and Relevance:**

In this diagnostic study of 2 ED eTrigger cohorts, model performance varied by cohort, with LLMs showing similar discrimination but different binary thresholds. These findings suggest that evaluation within the review workflow is needed before implementation and that reviewer-like concordance captures a distinct dimension of model behavior from discrimination.

## Key Points

**Question:**

Among 2 emergency department electronic trigger cohorts used for quality review, how did commercial large language models (LLMs) perform for identifying missed opportunities for diagnosis (MODs)?

**Findings:**

In this diagnostic study of 288 encounters, LLMs showed broadly similar discrimination for MODs but differing operating thresholds when asked to make binary adjudications, which were reflected in different physician-model concordances.

**Meaning:**

These findings suggest that LLM-based screening for MODs should be evaluated within the target clinical context and that physician-like concordance and adjudicated screening performance are distinct model properties.

## Teaser

This diagnostic study compares the ability of 6 different large language models (LLMs) to identify missed opportunities for diagnosis among patients admitted to emergency departments (EDs) that use electronic trigger (eTrigger) tools for quality and safety surveillance.

## Introduction

Diagnostic quality is a key objective of emergency departments (EDs), and missed opportunities for diagnosis (MODs) are an important cause of patient harm.^1,2^ Trigger tools are an established patient-safety surveillance method used to identify records of patients at increased risk of harm for focused review. In emergency care, this methodology has been adapted into ED-specific electronic trigger tools (eTriggers) for quality and safety surveillance and has evolved through consensus development, automated implementation, and multicenter validation.^3,4,5^ MOD detection and remediation are one diagnostic-safety application of this broader trigger-based review approach and typically require time-intensive expert medical record review.^6,7^ Although eTriggers enrich for cases at higher risk of diagnostic error, even these enriched cohorts have relatively low MOD yields.^6,8,9,10,11^

Large language models (LLMs) can rapidly synthesize clinical records and may assist with diagnostic safety review, but comparative evidence in real-world patient care datasets remains limited.^12,13,14,15,16,17^ In emergency care, emerging frameworks position artificial intelligence (AI) as a tool to support information gathering, clinical decision support, and quality-improvement feedback within human-centered workflows.^18^ Different models may also adopt different operating thresholds when asked to identify potential MODs, and their behavior may vary across clinical cohorts. Because deployment may depend not only on case-finding performance but also on how model outputs interface with physician review, it is useful to understand both aggregate performance against an adjudicated reference standard and concordance with individual physician reviewers.

In this study, we used 2 established ED eTrigger cohorts to build an evaluation dataset for comparing commercially available LLMs. We focused on 3 questions: (1) how models differed across screening measures (ie, sensitivity, specificity, positive predictive value [PPV] and negative predictive value [NPV]); (2) model discrimination as captured by area under the receiver operating characteristic curve (AUC); and (3) model alignment with individual physician reviewers as assessed using a reviewer-wise concordance analysis.

## Methods

### Study Design and Setting

This retrospective diagnostic study included encounters from 9 hospitals (2 academic and 7 community) across the Beth Israel Lahey Health enterprise between April 2015 and March 2025. We followed the Transparent Reporting of a Multivariable Prediction Model for Individual Prognosis or Diagnosis–LLM (TRIPOD-LLM) reporting guideline.^19^ Age, sex, race, and ethnicity were extracted from the electronic health record (EHR) registration record, where race and ethnicity are collected during patient registration. Race and ethnicity categories included American Indian or Alaska Native, Asian, Black or African American, Hispanic, White, other (any race or ethnicity not otherwise specified), and unable to obtain or unknown race; race and ethnicity were included to characterize the study population and to assess the generalizability of findings to other settings. The study was deemed exempt from review and the requirement of informed consent by the Beth Israel Deaconess Medical Center institutional review board because the study used retrospective data and did not affect clinical care.

### eTrigger Cohorts

eTriggers are automatable administrative criteria used to identify cases at increased risk for MODs. Based on prior literature and operational relevance, we used 2 established primary cohorts: ED discharge with return hospital admission within 72 hours and non–intensive care unit (ICU) admission with escalation to ICU-level care within 24 hours.^8,9,11,20^ For the 72-hour return cohort, we prespecified stratified random sampling from the academic medical center that was the primary study site (Beth Israel Deaconess Medical Center) and from the remainder of the network to permit descriptive stratum-specific assessment. The academic medical center joined the enterprise Epic in 2024, so all cases in that stratum are from 2024 or more recent. Similarly, during review we noted that ICU coding data in the EHR became more reliable as of 2024, so we limited the start of the floor-to-ICU cohort to that year. Eligible encounters were identified using custom SQL (structured query language) queries in the Enterprise Snowflake Data Warehouse (eMethods in Supplement 1).

### Sampling Strategy

The primary study objective was comparative evaluation rather than population-level trigger prevalence estimation. We targeted approximately 100 evaluable encounters for each of the two 72-hour returns-to-admission strata as well as the floor-to-ICU cohort, a pragmatic sample size for dual-physician adjudication and model comparison.^8^ For a proportion of 50%, corresponding to the maximum binomial variance, a sample size of 100 yields a 95% CI half-width of less than 10% for PPV or NPV using the Wilson score interval.

### Physician Adjudication of Evaluation Cases

All sampled cases were independently reviewed by 2 of 5 emergency physicians (C.M.M., D.S., S.G., B.S., and C.G.) using the question: “was there a missed opportunity to make a correct or timely diagnosis based on the available evidence, regardless of harm?” Reviewers assessed EHR documentation of the ED visit and subsequent hospitalization records assembled for each trigger cohort without restrictions on review content. Reviewers were provided structured instructions that incorporated the Safer Dx instrument (eMethods in Supplement 1) as a rubric for binary case-level adjudication rather than as a numeric score with a prespecified cutoff.^6^ A training subset was reviewed by all case reviewers to align the application of the rubric. Disagreements were adjudicated to establish a final case label, and persistent disagreements were resolved by an additional reviewer (A.D.H.). Cases later found not to meet inclusion criteria (eg, miscoded ICU escalation or absent ED documentation) were excluded from further analysis. Reviewers timed themselves on a subset of medical records, and median review time is reported.

### Model Evaluation

Prompt development followed a structured iterative process using Claude Sonnet 4. Initial prompts were adapted from human reviewer instructions and the Safer Dx Framework, and 2 physician authors (C.M.M. and A.D.H.) tested the prompt on small numbers of cases in each cohort, with wording refined to address recurring failure modes. Final prompts were locked before full-scale analysis; the same prompt and case-input assembly were applied across all evaluated models without model-specific revisions (eMethods in Supplement 1). Evaluated models were Claude Sonnet 4 (Anthropic), Claude Sonnet 4.6 (Anthropic), Claude Opus 4.6 (Anthropic), Gemini 3 Pro (Google DeepMind), GPT-5 (OpenAI), and GPT-5 mini (OpenAI), with last date of use in March 2026. Each model returned a structured JavaScript object notation containing a binary MOD classification (ie, yes or no), a MOD probability estimate from 0% to 100%, and a free-text rationale. Binary classifications were used for sensitivity, specificity, PPV, and NPV; probability estimates were used for AUC calculations. Claude Sonnet 4 was accessed through an enterprise-managed, protected health information–compliant Amazon Web Services entry point, and the remaining models were accessed through enterprise-managed Snowflake with model parameters and training cut-off dates described in eMethods in Supplement 1. Single-run inference was used.

### Statistical Analysis

For each cohort, we report the physician-adjudicated MOD yield (the sampled trigger PPV), the number needed to screen to identify 1 MOD, and screening minutes per detected MOD. As a primary analysis, we calculated sensitivity, specificity, PPV, and NPV from the binary MOD output with 95% CIs using Wilson score intervals. AUC was computed from model likelihood scores using the nonparametric Mann-Whitney formulation. The 95% CIs used the DeLong method. Exploratory, unadjusted pairwise comparisons of the highest observed AUC model with each of the remaining models within each cohort used the DeLong test for correlated receiver operating characteristic curves.^21^ We estimated potential reviewer time savings in each cohort by identifying the most restrictive per-model MOD likelihood score threshold that maintained at least 80% sensitivity performance, then comparing the resulting number of records requiring physician review with review of all records.

As a secondary analysis, we assessed which models were most physician-like in their MOD assessments. Here, we calculated reviewer-reviewer and reviewer-model percentage agreement and Gwet AC1 across all cases, limiting reviewer-reviewer pairwise comparisons to those with a minimum of 20 observations.^22^ The 95% CIs for these concordance measures were obtained by percentile bootstrap with 2000 replicates. For each model, we then calculated the mean reviewer-model Gwet AC1 across the physicians it was compared against. Differences between the top model and the other models on this mean reviewer-model Gwet AC1 metric were evaluated using 2000 paired bootstraps, with statistical significance inferred when the 95% CI for the difference excluded 0. Analyses were performed using Python version 3.11 (Python Software Foundation). Google Gemini and OpenAI Codex were used to assist with SQL and Python code development. Code is available on request.

## Results

**Figure 1.**
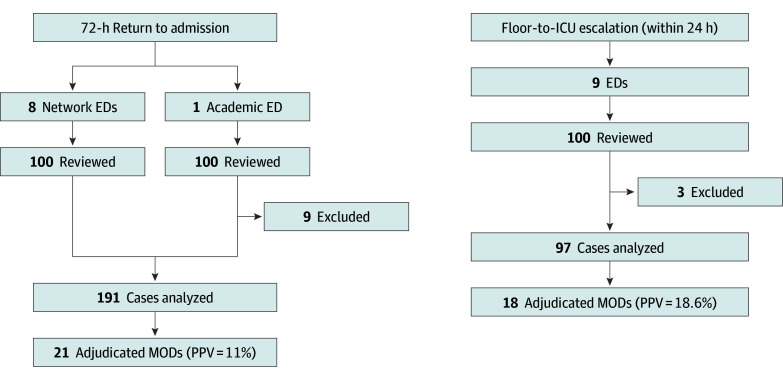
Study Flow Diagram The 2 flow diagrams show study flow for 72-hour return to admission and floor-to–intensive care unit (ICU) escalations. ED indicates emergency department; MOD, missed opportunities for diagnosis; PPV, positive predictive value.

Among 300 sampled primary-cohort encounters, 12 were excluded, including 9 duplicate 72-hour return encounters and 3 floor-to-ICU cases found on review to be miscoded, leaving 288 analyzed encounters with 39 MODs (13.5%). The analyzed cohort had a median (IQR) age of 69 (54-79) years, 135 patients (46.9%) were female, 191 encounters were 72-hour return admissions, and 97 were floor-to-ICU escalations (Table 1, Figure 1, and eTable 1 in Supplement 1). Among 72-hour return admissions, the academic stratum (98 cases) was entirely from 2024 onward, whereas 24.7% of the network stratum (23 of 93 cases) was from 2024 onward (eTable 1 in Supplement 1). In this cohort, 21 cases (11.0%) were adjudicated as MODs, corresponding to a trigger PPV of 11.0% and a number needed to screen of 9.1. In the floor-to-ICU cohort, 18 cases (18.6%) were adjudicated as MODs, corresponding to a trigger PPV of 18.6% and a number needed to screen of 5.4. Median (IQR) physician review time was 5 (3-7) minutes per case, yielding 45.5 minutes per detected MOD in 72-hour return cases and 26.9 minutes per detected MOD in floor-to-ICU cases. Across all cases, reviewer agreement was 81.9% (95% CI, 77.4%-86.1%), with Gwet AC1 of 0.77 (95% CI, 0.70-0.83).

**Table 1.** Study Demographics

Characteristic	Participants, No. (%)		
	Overall (N = 288)	72-h Return to admission (n =191)	Floor to intensive care unit within 24 h (n = 97)
Age, median (IQR), y	69 (54-79)	65 (51-78)	71 (59-80)
Sex			
Female	135 (46.9)	95 (49.7)	40 (41.2)
Male	153 (53.1)	96 (50.3)	57 (58.8)
Race			
American Indian or Alaska Native	1 (0.3)	1 (0.5)	0
Asian	5 (1.7)	3 (1.6)	2 (2.1)
Black or African American	27 (9.4)	23 (12.0)	4 (4.1)
White	238 (82.6)	149 (78.0)	89 (91.8)
Patient chose not to disclose	4 (1.4)	3 (1.6)	1 (1.0)
Unable to obtain	1 (0.3)	1 (0.5)	0
Other^a^	11 (3.8)	10 (5.2)	1 (1.0)
Ethnicity			
Hispanic	17 (5.9)	14 (7.3)	3 (3.1)
Non-Hispanic	271 (94.1)	177 (92.7)	94 (96.9)

^a^ The other category was obtained directly from the electronic health record and includes any race not otherwise specified.

**Figure 2.**
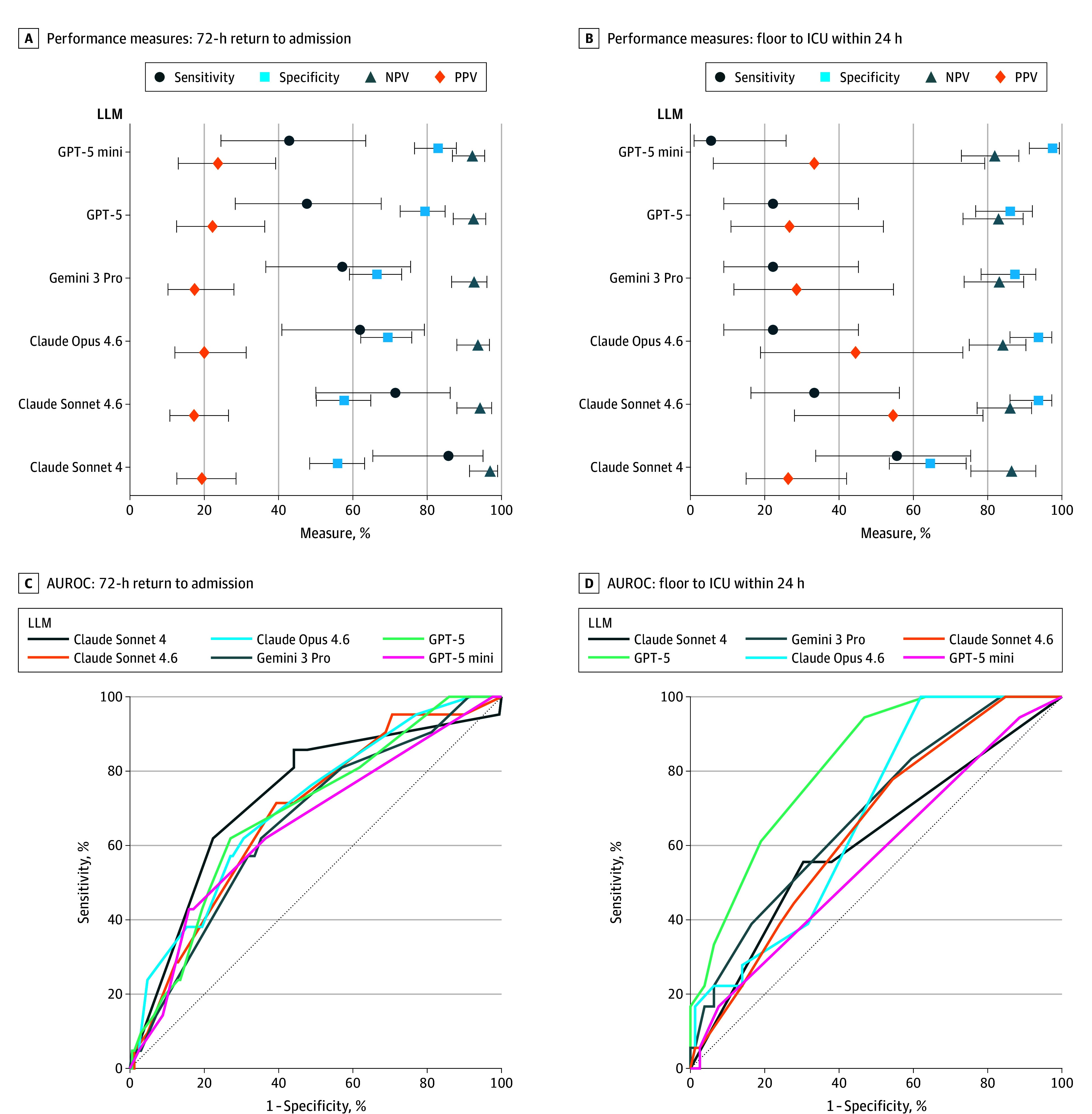
Dot Plots and Line Graphs of Performance of Large Language Models (LLMs) for Identifying Diagnostic Error in 2 Emergency Care Cohorts Panels A and B show sensitivity, specificity, negative predictive value (NPV), and positive predictive value (PPV), with 95% CIs (error bars), for the 72-hour return to hospital admission cohort and the floor-to–intensive care unit (ICU) transfer within 24 hours cohort, respectively. Panels C and D show area under the receiver operating characteristic curves (AUROCs) for the same models in each cohort. All performance measures were calculated against physician-adjudicated reference standard classifications. Confusion matrices and specific values are in eTable 2 and eTable 3 in Supplement 1.

In the 72-hour return cohort, model performance reflected a sensitivity-specificity tradeoff (Figure 2, Table 2, and eTables 2-3 in Supplement 1). Claude Sonnet 4 had the highest sensitivity (85.7%; 95% CI, 65.4%-95.0%) and lowest specificity (55.9%; 95% CI, 48.4%-63.1%), whereas GPT-5 mini was the most conservative, with the lowest sensitivity (42.9%; 95% CI, 24.5%-63.5%) and highest specificity (82.9%; 95% CI, 76.6%-87.9%). AUCs were broadly similar across models, ranging from 0.65 (95% CI, 0.53-0.77) for GPT-5 mini to 0.73 (95% CI, 0.61-0.85) for Claude Sonnet 4. Exploratory paired comparisons between Claude Sonnet 4 and the remaining models were not significant (eTable 4 in Supplement 1). Model ordering was directionally similar in the academic and network strata (eFigure 1 in Supplement 1). Claude Sonnet 4 had the most substantial time-savings while maintaining 80% sensitivity with 99 of 191 cases (51.8%) screened out with a resultant 8.2-hour time-savings assuming a 5-minute review per case (eTable 5 in Supplement 1).

**Table 2.** LLM Performance by Cohort

LLM	Sensitivity, % (95% CI)	Specificity, % (95% CI)	PPV, % (95% CI)	NPV, % (95% CI)	AUC (95% CI)
72-h Return to admission (n = 191)					
Claude Sonnet 4	85.7 (65.4-95.0)	55.9 (48.4-63.1)	19.4 (12.6-28.5)	96.9 (91.4-99.0)	0.73 (0.61-0.85)
Claude Sonnet 4.6	71.4 (50.0-86.2)	57.6 (50.1-64.8)	17.2 (10.7-26.5)	94.2 (88.0-97.3)	0.68 (0.57-0.80)
Claude Opus 4.6	47.6 (28.3-67.6)	79.4 (72.7-84.8)	22.2 (12.5-36.3)	92.5 (87.0-95.7)	0.69 (0.57-0.80)
Gemini 3 Pro	61.9 (40.9-79.2)	69.4 (62.1-75.8)	20.0 (12.1-31.3)	93.7 (88.0-96.7)	0.70 (0.59-0.82)
GPT-5	57.1 (36.5-75.5)	66.5 (59.1-73.1)	17.4 (10.2-28.0)	92.6 (86.6-96.1)	0.66 (0.54-0.78)
GPT-5 mini	42.9 (24.5-63.5)	82.9 (76.6-87.9)	23.7 (13.0-39.2)	92.2 (86.8-95.5)	0.65 (0.53-0.77)
Floor to intensive care unit within 24 h (n = 97)					
Claude Sonnet 4	55.6 (33.7-75.4)	64.6 (53.6-74.2)	26.3 (15.0-42.0)	86.4 (75.5-93.0)	0.61 (0.47-0.75)
Claude Sonnet 4.6	22.2 (9.0-45.2)	86.1 (76.8-92.0)	26.7 (10.9-52.0)	82.9 (73.4-89.5)	0.65 (0.52-0.77)
Claude Opus 4.6	22.2 (9.0-45.2)	87.3 (78.2-93.0)	28.6 (11.7-54.6)	83.1 (73.7-89.7)	0.68 (0.56-0.79)
Gemini 3 Pro	22.2 (9.0-45.2)	93.7 (86.0-97.3)	44.4 (18.9-73.3)	84.1 (75.0-90.3)	0.69 (0.56-0.81)
GPT-5	33.3 (16.3-56.3)	93.7 (86.0-97.3)	54.5 (28.0-78.7)	86.0 (77.2-91.8)	0.82 (0.73-0.91)
GPT-5 mini	5.6 (1.0-25.8)	97.5 (91.2-99.3)	33.3 (6.1-79.2)	81.9 (72.9-88.4)	0.57 (0.46-0.67)

Abbreviations: AUC, area under the receiver operating characteristic curve; LLM, large language model; NPV, negative predictive value; PPV, positive predictive value.

In the floor-to-ICU cohort, performance shifted toward lower sensitivity and higher specificity for most models (Figure 2, Table 2, and eTables 2-4 in Supplement 1). Sensitivity ranged from 5.6% (95% CI, 1.0-25.8%) for GPT-5 mini to 55.6% (95% CI, 33.7%-75.4%) for Claude Sonnet 4, and specificity ranged from 64.6% (53.6%-74.2%) for Claude Sonnet 4 to 97.5% (95% CI, 91.2%-99.3%) for GPT-5 mini. GPT-5 had the highest observed AUC at 0.82 (95% CI, 0.73-0.91), while GPT-5 mini had the lowest AUC at 0.57 (95% CI, 0.46-0.67). In paired comparisons, GPT-5 had a higher AUC than the other tested models (eTable 4 in Supplement 1) and also had the most substantial time-savings while maintaining 80% sensitivity with 43 of 97 cases (44.3%) screened out with a resulting 3.6-hour time-savings (eTable 5 in Supplement 1).

**Figure 3.**
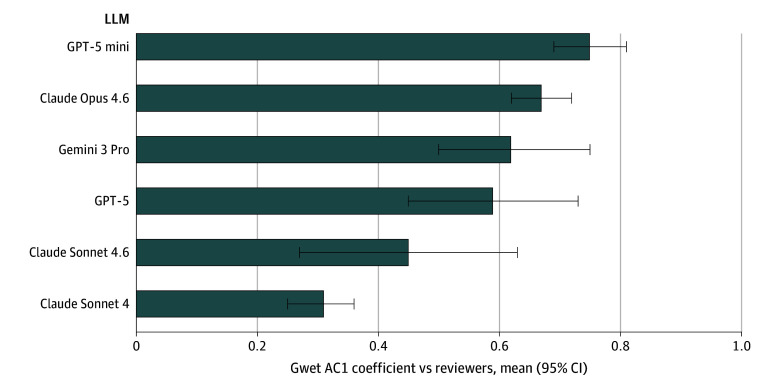
Concordance Between Each Large Language Model (LLM) and Physician Reviewers Bars show the mean Gwet AC1 coefficient for each model across reviewer-model comparisons in the pooled 72-hour return to admission and floor-to-intensive care unit transfer cohorts. Error bars indicate 95% CIs. Higher values indicate greater agreement with physician reviewer classifications.

In the exploratory concordance analysis, physician-to-physician agreement across the 5 reviewers (4 reviewer pairs) ranged from 76.6% (95% CI, 67.5%-85.7%) to 92.6% (95% CI, 85.2%-98.2%), with Gwet AC1 values ranging from 0.68 (95% CI, 0.52-0.82) to 0.91 (95% CI, 0.80-0.98) (eFigure 2 in Supplement 1). Mean physician-to-physician Gwet AC1 was 0.78 (95% CI, 0.70-0.86). Each model was then compared with each of the 5 physicians on the cases reviewed by that physician. Across models, mean physician-model Gwet AC1 ranged from 0.31 (95% CI, 0.25-0.36) for Claude Sonnet 4 to 0.75 (95% CI, 0.69-0.81) for GPT-5 mini (Figure 3 and eFigure 2 in Supplement 1).

## Discussion

In this diagnostic study of 2 established ED eTrigger cohorts, commercially available LLMs displayed differing performance across settings. We observed that similar model AUCs did not translate to similar binary case determinations; similar threshold-related behavior has been observed in real-world ED note evaluations, where LLMs tended to make overly cautious recommendations with relatively high sensitivity at the expense of specificity.^23^ Across all cases, LLM review would have reduced single-physician review effort from 24 hours to 12.2 hours, for a total savings of 11.8 hours while maintaining greater than 80% sensitivity.

Our data address an important gap in the medical LLM literature. A recent systematic review suggests that most health care LLM evaluations have relied on examination-style or otherwise nonclinical benchmarks, with only 5% using real patient care data and few assessing deployment considerations or calibration.^24^ In contrast, this study evaluates models in adjudicated, operational diagnostic-safety cohorts derived from routine emergency care, which reflects how such systems may be used in practice.

Our findings suggest that commercially available LLMs are best used as clinician-facing prescreening rather than as stand-alone MOD adjudicators; the appropriate operating characteristics will largely depend on the workflow in which these models are implemented. For example, a program seeking a sensitive initial screen may prefer a different operating point than one seeking a narrower, higher-yield review queue, and the preferred tradeoff may differ across populations. Consistent with emerging emergency medicine and health system implementation frameworks, local benchmarking, prospective validation, and ongoing monitoring are needed before routine use.^18^

MOD detection has traditionally been retrospectively adjudicated with standardized frameworks. However, this study offers new opportunities for site- and context-specific adjudication. While large, curated fine-tuning datasets for diagnostic quality review are difficult to assemble, our findings suggest that performance may depend as much on prompt and workflow design as on the underlying base model. A near-term strategy may therefore be stakeholder-guided prompt optimization: iteratively refining instructions, case packaging, output structure, and decision thresholds using clinician review of failure cases and typical workflow constraints. Human-in-the-loop prompt optimization approaches, such as interactive prompt optimization frameworks that allow users to inspect prompt variants, informative cases, model predictions, explanations, and performance metrics, may offer one practical model for this type of development.^25,26^

Fundamentally, implementing MOD detection with LLMs into a quality framework is a human-centered design problem.^27^ Prior implementation literature suggests that successful clinical AI tools depend not only on technical accuracy but also on workflow fit, usability, trust, transparency, and stakeholder involvement.^28,29^ In this context, reviewer-model concordance is not interchangeable with adjudicated discrimination, but it may capture a complementary and implementation-relevant dimension of model behavior: whether outputs are sufficiently aligned with physician review patterns to be usable in practice.^30^ Future work should therefore combine adjudicated benchmarking with stakeholder-guided prompt iteration, local calibration, silent prospective validation, and direct evaluation of reviewer workload, trust, case yield, and adoption.^18,24^

### Limitations

This study has several limitations. First, cases came from a single health system, which may limit generalizability to other documentation environments, patient populations, and review practices. Second, all models were evaluated with a single prompt and without model-specific tuning; relative performance in deployment could differ after prompt or threshold optimization. Third, although we attempted to provide models with the clinical information most relevant to diagnostic review, physician reviewers may have considered portions of the EHR not included in the model input, which could have influenced adjudication and model-reviewer comparisons. Fourth, there is possibility of temporal performance bias because most of the 72-hour return network stratum cases were from before 2024. Fifth, the evaluation focused on binary MOD adjudication and did not evaluate severity, downstream harm, or diagnostic error subtype. Sixth, the reviewer-concordance analysis included only 5 physicians and should be interpreted as exploratory.

## Conclusions

In this diagnostic study across 2 ED cohorts, commercially available LLMs showed meaningful differences in default threshold behavior when screening for MODs. These findings support cohort-specific benchmarking and threshold calibration before clinical use and suggest that reviewer-like concordance should be interpreted as complementary to, rather than a substitute for, adjudicated screening performance.

## Notes

Supplement 1. eMethods. eTable 1. Strata demographics in 72-hour return cohort: Academic medical center vs network eTable 2. Full confusion matrix for all LLMs eTable 3. All model performance findings with 95% confidence intervals eTable 4. Pairwise DeLong comparisons of the highest-observed AUC model versus the remaining models within each cohort eFigure 1. Model performance across academic and network strata for ED returns to admission within 72 hours eTable 5. Estimated single-reviewer chart-screening savings for all models at 80% sensitivity eFigure 2. Pairwise agreement among physician reviewers and large language models across adjudicated emergency care cases

Supplement 2. Data Sharing Statement
