# Supplementary material for: Screening for Missed Opportunities for Diagnosis in the ED Using eTriggers and Large Language Models
Source: JAMA Netw Open. 2026 Jun 29;9(6):e2620939. doi: 10.1001/jamanetworkopen.2026.20939 (PMC13316604; doi:10.1001/jamanetworkopen.2026.20939)

Supplemental Online Content

Marks CM, Gibney S, Stenson B, et al. Screening for missed opportunities for diagnosis in the ED using eTriggers and large language models. *JAMA Netw. Open*. 2026;9(6):e2620939. doi:10.1001/jamanetworkopen.2026.20939

eMethods.
eTable 1. Strata demographics in 72-hour return cohort: Academic medical center vs network
eTable 2. Full confusion matrix for all LLMs
eTable 3. All model performance findings with 95% confidence intervals
eTable 4. Pairwise DeLong comparisons of the highest-observed AUC model versus the remaining models within each cohort
eFigure 1. Model performance across academic and network strata for ED returns to admission within 72 hours
eTable 5. Estimated single-reviewer chart-screening savings for all models at 80% sensitivity
eFigure 2. Pairwise agreement among physician reviewers and large language models across adjudicated emergency care cases

This supplemental material has been provided by the authors to give readers additional information about their work.

eMethods.

eTrigger SQL criteria

1. 72 hour return to admission:
    a. Inclusion Criteria
        i. Patients with at least two emergency department (ED) encounters.
        ii. The first (index) ED encounter must meet all of the following:
            1. ED disposition is "Discharge".
        iii. The second (return) ED encounter must meet all of the following:
            1. ED disposition is "Admit".
            2. A bed request was placed.
            3. Arrival time is after the departure time of the first encounter.
            4. The number of days between the first departure and second arrival is between 1 and 3, inclusive.
    b. Exclusion Criteria
        i. The second encounter has levelofcare = 'Behavioral Health'.
        ii. The department specialty of the first encounter begins with "Urgent Care".

2. Floor-to-ICU escalation within 24 hours:
    a. Inclusion criteria
        i. The first (earliest) uncancelled bed request (if present) is not for Intensive Care level of care.
        ii. There is a subsequent uncanceled bed request for Intensive Care level of care.
        iii. The Intensive Care bed request occurs within 24 hours of ED arrival
        iv. The Intensive Care bed request is the first such request for the encounter (i.e., it is the first non-canceled ICU request by request time).
    b. Exclusion criteria
        i. Encounters where no ICU bed request was made.
        ii. Encounters where the ICU bed request occurred more than 24 hours after ED arrival.

iii. Encounters where the first uncanceled bed request was already for Intensive Care (i.e., not a transition from a lower level of care).

Reviewer MOD criteria

Reviewer Missed Opportunity for Diagnosis (MOD) criteria

For each ED encounter, was there a missed opportunity to make a correct or timely diagnosis based on the available evidence, regardless of harm? This standard is asking whether a perfect emergency physician operating at their best could have made this diagnosis, not whether we are sure we would have done so 100% of the time. For an MOD to have occurred, the patient should have signs, symptoms, laboratory or imaging findings suggestive of an alternate diagnosis that could have been made by the emergency physician.

Example: MOD

1. Patient with diabetes with lower extremity weakness is admitted to medicine for workup of weakness. ED labs with elevated anion gap and low bicarbonate consistent with euglycemic DKA, without specific mention or action around this diagnosis.

Example: not an MOD

1. Patient presents with new onset confusion and erratic behavior. Labs and CT are normal. Patient is admitted to medicine with AMS. During hospitalization, the patient is diagnosed with anti-NMDA receptor encephalitis. *Reason*: Signs and symptoms were present at time of evaluation, but this is not a diagnosis that can be made by an emergency physician as the LP results would not be available during the encounter.
2. Patient presents with back pain after lifting weights. No fevers, IV drug use, or other risk factors noted. Patient returns in 1 week with fever and back pain, found to have spinal epidural abscess. *Reason:* The signs and symptoms at time of evaluation were not suggestive of SEA as the diagnosis.

The following factors are associated with the presence of a missed opportunity for diagnosis (Recommendations for using the Revised Safer Dx Instrument to help measure and improve diagnostic safety. Diagnosis (Berl). 2019;6(4):315-323, see graphic below).

The Safer Dx Instrument: Items for Determining Presence or Absence of a Diagnostic Missed Opportunity

Rate the following items for the episode of care under review:
1—2—3—4—5—6—7
1 = Strongly Disagree 7 = Strongly Agree

Item		Score
1.	The documented history was suggestive of an alternate diagnosis, which was not considered in the diagnostic process.	
2.	The documented physical exam was suggestive of an alternate diagnosis, which was not considered in the diagnostic process.*	
3.	Data gathering through history, physical exam, and review of prior documentation (including prior laboratory, radiology, pathology or other results) was incomplete, given the patient's medical history and clinical presentation.	
4.	Alarm symptoms or "Red Flags" (i.e. features in the clinical presentation that are considered to predict serious disease) were not acted upon.	
5.	The diagnostic process was affected by incomplete or incorrect clinical information given to the care team by the patient or their primary caregiver.	
6.	The clinical information (i.e. history, physical exam or diagnostic data) should have prompted additional diagnostic evaluation through tests or consults.	
7.	The diagnostic reasoning was not appropriate, given the patient's medical history and clinical presentation.	
8.	Diagnostic data (laboratory, radiology, pathology or other results) available or documented were misinterpreted in relation to the subsequent final diagnosis.	
9.	There was missed follow-up of available or documented diagnostic data (laboratory, radiology, pathology or other results) in relation to the subsequent final diagnosis.	
10.	The differential diagnosis was not documented OR The documented differential diagnosis did not include the subsequent final diagnosis.	
11.	The final diagnosis was not an evolution of the care team's initial presumed diagnosis (or working diagnosis).	
12.	The clinical presentation at the initial or subsequent presentation was mostly typical of the final diagnosis.	
13.	In conclusion, based on all the above questions, the episode of care under review has a missed opportunity to make a correct and timely diagnosis.	

* Physical exam includes vital signs

Additional information, please check "Yes" if applicable:

1. Care episode involves a management error. ☐ Yes
2. Care escalation (e.g. hospitalization at subsequent visit) was related to worsening of an original correctly diagnosed condition that the patient initially presented with (rather than from something being missed initially) ☐ Yes
3. Patient initially refused admission or additional evaluation. ☐ Yes

LLM MOD review prompt

You are ED-Diagnostic-Error-Review-Bot, reasoning like an expert emergency physician.

Task: do *all* of the following:

1. **Read every data block once, think, then decide** whether the first ED visit contained a *missed opportunity for diagnosis* (MOD) considering
   - the 13 items of the **SaferDx Instrument**
   - the MOD definition & examples given below (*built-in for your reference—do not quote back*).
2. Provide a likelihood estimate (0-100%) that there was a missed opportunity for diagnosis. You are NOT to consider management, only diagnosis.
3. Make a **Yes/No call**:
   - **Yes** if your clinical judgment clearly supports a missed opportunity.
   - **No** otherwise.
4. List up to 5 details that anchor your determination
5. Output a JSON and nothing else:

'''
{{
  "mod_decision": "Yes", // or "No"
  "mod_likelihood_estimate": "0-100%"
  "mod_rationale": "Brief narrative summary of why MOD is or is not present, focused on diagnostic reasoning."
}}
'''

Here are the SaferDx criteria to consider:

1. The documented history was suggestive of an alternate diagnosis, which was not considered in the diagnostic process.
2. The documented physical exam was suggestive of an alternate diagnosis, which was not considered in the diagnostic process.
3. Data gathering through history, physical exam, and review of prior documentation (including prior laboratory, radiology, pathology, or other results) was incomplete, given the patient's medical history and clinical presentation.
4. Alarm symptoms or "red flags" (i.e., features in the clinical presentation that are considered to predict serious disease) were not acted upon.
5. The diagnostic process was affected by incomplete or incorrect clinical information given to the care team by the patient or their primary caregiver.
6. The clinical information (i.e., history, physical exam or diagnostic data) should have prompted additional diagnostic evaluation through tests or consults.
7. The diagnostic reasoning was not appropriate, given the patient's medical history and clinical presentation.
8. Diagnostic data (laboratory, radiology, pathology or other results) were available or documented were misinterpreted in relation to the subsequent final diagnosis.
9. There was missed follow-up of available or documented diagnostic data (laboratory, radiology, pathology or other results) in relation to the subsequent final diagnosis.
10. The differential diagnosis was not documented OR the documented differential diagnosis did not include the subsequent final diagnosis.
11. The final diagnosis was not an evolution of the care team's initial presumed diagnosis (or working diagnosis).
12. The clinical presentation at the initial or subsequent presentation was mostly typical of the final diagnosis or more amenable to a correct and timely diagnosis.
13. In conclusion, based on all the above questions, the episode of care under review has a missed opportunity to make a correct and timely diagnosis.

For each ED encounter, was there a missed opportunity to make a correct or timely diagnosis based on the available evidence, regardless of harm? This standard is asking whether a perfect

emergency physician operating at their best could have made this diagnosis, not whether we are sure we would have done so 100% of the time. For an MOD to have occurred, the patient should have signs, symptoms, laboratory or imaging findings suggestive of an alternate diagnosis that could have been made by the emergency physician.

Examples:

MOD:

Patient with diabetes with lower extremity weakness is admitted to medicine for workup of weakness. ED labs with elevated anion gap and low bicarbonate consistent with euglycemic DKA, without specific mention or action around this diagnosis.

Not an MOD:

Patient presents with new onset confusion and erratic behavior. Labs and CT are normal. Patient is admitted to medicine with AMS. During hospitalization, the patient is diagnosed with anti-NMDA receptor encephalitis. Reason: Signs and symptoms were present at time of evaluation, but this is not a diagnosis that can be made by emergency physician as the LP results would not be available during the encounter.

Patient presents with back pain after lifting weights. No fevers, IV drug use, or other risk factors noted. Patient returns in 1 week with fever and back pain, found to have spinal epidural abscess. Reason: The signs and symptoms at time of evaluation were not suggestive of SEA as the diagnosis.

Case Documentation
{case_details}

Output JSON:

Included clinical documentation for each of the eTrigger prompts

72 hour return to admission

- Encounter 1:
    - ED physician notes
    - ED nursing flowsheets (vital signs, nursing documentation)
    - Non-ED documentation (e.g., case management notes, consults)
    - ED labs - all labs resulting prior to patient departure
    - ED imaging reports as resulted prior to patient departure
- Encounter 2:
    - ED physician notes
    - ED nursing flowsheets (vital signs, nursing documentation)
    - Non-physician documentation (e.g., case management notes, consults)
    - Notes from first 48 hours of hospitalization with exclusions*

Floor admission with ICU escalation within 24 hours:

- ED physician notes
- ED nursing flowsheets (vital signs, nursing documentation)
- Non-ED documentation (e.g., case management notes, consults) within 48 hours with exclusions*
- ED labs - all labs resulting prior to patient departure
- ED imaging reports as resulted prior to patient departure

* Excluded note types:

ACP (Advance Care Planning)

Brief Op Note

Care Coordination Note

Code Documentation

Code Status and Healthcare Proxy

Diabetic Education

Discharge Instructions

Discharge Instr - Activity

Discharge Instr - AVS First Page
Discharge Instr - Diet
Discharge Instr - Lab
Discharge Instr - Other Orders
Discharge Instr - Pt Ed Handouts
Discharge Instr-Facility, Services & DME
Discharge Instr - Wound
Downtime Event Note
ED AVS Snapshot
ED Information Exchange
ED Procedure Note
Global ED Visit
H&P (View-Only)
Interval H&P Note
IP AVS Snapshot
IV Therapy Notes
Liaison Communication
Letter
Medical Student
Moderate Sedation
NCDR Registry
OR Nursing
Op Note
Patient Care Conference
PCU Note
Research
Treatment Plan
Utilization Review
Wound Ostomy Note

Model parameters:

	Reasoning	Temperature	Checkpoints
**GPT-5 mini**	Minimal	0	2025-08-07
**GPT-5**	Minimal	0	2024-09-30
**Gemini 3 Pro**	Low	0	2025-01
**Claude Sonnet 4.6**	None	0	2025-05
**Claude Opus 4.6**	None	0	2025-05
**Claude Sonnet 4**	None	0.1	2025-05-14

eTable 1. Strata demographics in 72-hour return cohort: Academic medical center vs network			
**Characteristic**	**Overall**	**Academic medical center**	**Network**
N	191	98	93
% of cases since 2024	63.4%	100.0%	24.7%
Age, median [IQR]	65 [51, 78]	66 [52, 77]	65 [51, 80]
**Sex**			
Female	95 (49.7%)	48 (49.0%)	47 (50.5%)
Male	96 (50.3%)	50 (51.0%)	46 (49.5%)
**Race**			
White or Caucasian	149 (78.0%)	66 (67.3%)	83 (89.2%)
Black or African American	23 (12.0%)	21 (21.4%)	2 (2.2%)
Asian	3 (1.6%)	0 (0.0%)	3 (3.2%)
American Indian or Alaska Native	1 (0.5%)	0 (0.0%)	1 (1.1%)
Hispanic	1 (0.5%)	0 (0.0%)	1 (1.1%)
Patient chooses not to disclose	3 (1.6%)	3 (3.1%)	0 (0.0%)
Unable to Obtain	1 (0.5%)	1 (1.0%)	0 (0.0%)
Other	10 (5.2%)	7 (7.1%)	3 (3.2%)
**Ethnicity**			
Hispanic	14 (7.3%)	8 (8.2%)	6 (6.5%)
Non-Hispanic	177 (92.7%)	90 (91.8%)	87 (93.5%)

eTable 2. Full confusion matrix for all LLMs

72-hour return to admission (n = 191, 21 MODs)

Model	MODs identified	True positives	False positives	True negatives	False negatives
Claude Sonnet 4	93	18	75	95	3
Claude Sonnet 4.6	87	15	72	98	6
Claude Opus 4.6	45	10	35	135	11
Gemini 3 Pro	65	13	52	118	8
GPT-5	69	12	57	113	9
GPT-5 mini	38	9	29	141	12

Floor-to-ICU within 24 hours (n = 97, 18 MODs)

Model	MODs identified	True positives	False positives	True negatives	False negatives
Claude Sonnet 4	38	10	28	51	8
Claude Sonnet 4.6	15	4	11	68	14
Claude Opus 4.6	14	4	10	69	14
Gemini 3 Pro	9	4	5	74	14
GPT-5	11	6	5	74	12
GPT-5 mini	3	1	2	77	17

eTable 3. All model performance findings with 95% confidence intervals

72-hour returns to admission (n = 191)

Model	Sensitivity % (95% CI)	Specificity % (95% CI)	PPV % (95% CI)	NPV % (95% CI)	AUC (95% CI)
Claude Sonnet 4	85.7 (65.4-95.0)	55.9 (48.4-63.1)	19.4 (12.6-28.5)	96.9 (91.4-99.0)	0.73 (0.61-0.85)
Claude Sonnet 4.6	71.4 (50.0-86.2)	57.6 (50.1-64.8)	17.2 (10.7-26.5)	94.2 (88.0-97.3)	0.68 (0.57-0.80)
Claude Opus 4.6	47.6 (28.3-67.6)	79.4 (72.7-84.8)	22.2 (12.5-36.3)	92.5 (87.0-95.7)	0.69 (0.57-0.80)
Gemini 3 Pro	61.9 (40.9-79.2)	69.4 (62.1-75.8)	20.0 (12.1-31.3)	93.7 (88.0-96.7)	0.70 (0.59-0.82)
GPT-5	57.1 (36.5-75.5)	66.5 (59.1-73.1)	17.4 (10.2-28.0)	92.6 (86.6-96.1)	0.66 (0.54-0.78)
GPT-5 mini	42.9 (24.5-63.5)	82.9 (76.6-87.9)	23.7 (13.0-39.2)	92.2 (86.8-95.5)	0.65 (0.53-0.77)

Floor-to-ICU within 24 hours (n = 97)

Model	Sensitivity % (95% CI)	Specificity % (95% CI)	PPV % (95% CI)	NPV % (95% CI)	AUC (95% CI)
Claude Sonnet 4	55.6 (33.7-75.4)	64.6 (53.6-74.2)	26.3 (15.0-42.0)	86.4 (75.5-93.0)	0.61 (0.47-0.75)
Claude Sonnet 4.6	22.2 (9.0-45.2)	86.1 (76.8-92.0)	26.7 (10.9-52.0)	82.9 (73.4-89.5)	0.65 (0.52-0.77)
Claude Opus 4.6	22.2 (9.0-45.2)	87.3 (78.2-93.0)	28.6 (11.7-54.6)	83.1 (73.7-89.7)	0.68 (0.56-0.79)
Gemini 3 Pro	22.2 (9.0-45.2)	93.7 (86.0-97.3)	44.4 (18.9-73.3)	84.1 (75.0-90.3)	0.69 (0.56-0.81)
GPT-5	33.3 (16.3-56.3)	93.7 (86.0-97.3)	54.5 (28.0-78.7)	86.0 (77.2-91.8)	0.82 (0.73-0.91)
GPT-5 mini	5.6 (1.0-25.8)	97.5 (91.2-99.3)	33.3 (6.1-79.2)	81.9 (72.9-88.4)	0.57 (0.46-0.67)

72-hour returns to admission - academic strata (n = 98)

Model	Sensitivity % (95% CI)	Specificity % (95% CI)	PPV % (95% CI)	NPV % (95% CI)	AUC (95% CI)
Claude Sonnet 4	75.0 (46.8-91.1)	50.0 (39.7-60.3)	17.3 (9.4-29.7)	93.5 (82.5-97.8)	0.64 (0.45-0.82)
Claude Sonnet 4.6	75.0 (46.8-91.1)	58.1 (47.6-68.0)	20.0 (10.9-33.8)	94.3 (84.6-98.1)	0.69 (0.54-0.85)
Claude Opus 4.6	50.0 (25.4-74.6)	83.7 (74.5-90.0)	30.0 (14.5-51.9)	92.3 (84.2-96.4)	0.73 (0.59-0.87)
Gemini 3 Pro	58.3 (32.0-80.7)	67.4 (57.0-76.4)	20.0 (10.0-35.9)	92.1 (82.7-96.6)	0.66 (0.51-0.81)

GPT-5	58.3 (32.0-80.7)	66.3 (55.8-75.4)	19.4 (9.8-35.0)	91.9 (82.5-96.5)	0.67 (0.50-0.83)
GPT-5 mini	41.7 (19.3-68.0)	82.6 (73.2-89.1)	25.0 (11.2-46.9)	91.0 (82.6-95.6)	0.65 (0.48-0.81)

**72-hour returns to admission - network strata (n = 93)**					
**Model**	**Sensitivity % (95% CI)**	**Specificity % (95% CI)**	**PPV % (95% CI)**	**NPV % (95% CI)**	**AUC (95% CI)**
Claude Sonnet 4	100.0 (70.1-100.0)	61.9 (51.2-71.6)	22.0 (12.0-36.7)	100.0 (93.1-100.0)	0.84 (0.76-0.93)
Claude Sonnet 4.6	66.7 (35.4-87.9)	57.1 (46.5-67.2)	14.3 (6.7-27.8)	94.1 (84.1-98.0)	0.68 (0.51-0.85)
Claude Opus 4.6	44.4 (18.9-73.3)	75.0 (64.8-83.0)	16.0 (6.4-34.7)	92.6 (83.9-96.8)	0.64 (0.44-0.84)
Gemini 3 Pro	66.7 (35.4-87.9)	71.4 (61.0-80.0)	20.0 (9.5-37.3)	95.2 (86.9-98.4)	0.75 (0.56-0.94)
GPT-5	55.6 (26.7-81.1)	66.7 (56.1-75.8)	15.2 (6.7-30.9)	93.3 (84.1-97.4)	0.65 (0.47-0.83)
GPT-5 mini	44.4 (18.9-73.3)	83.3 (73.9-89.8)	22.2 (9.0-45.2)	93.3 (85.3-97.1)	0.65 (0.46-0.84)

eTable 4. Pairwise DeLong comparisons of the highest-observed AUC model versus the remaining models within each cohort

**72-hour return to admission: Claude Sonnet 4 had the highest observed AUC (0.73)**					
**Top observed AUC model**	**Top model AUC (95% CI)**	**Comparator**	**Comparator AUC (95% CI)**	**Delta AUC (95% CI)**	**Paired DeLong P value**
Claude Sonnet 4	0.73 (0.61-0.85)	GPT-5 mini	0.65 (0.53-0.77)	0.08 (-0.05-0.20)	0.235
Claude Sonnet 4	0.73 (0.61-0.85)	GPT-5	0.66 (0.54-0.78)	0.07 (-0.05-0.19)	0.263
Claude Sonnet 4	0.73 (0.61-0.85)	Claude Sonnet 4.6	0.68 (0.57-0.80)	0.05 (-0.07-0.16)	0.453
Claude Sonnet 4	0.73 (0.61-0.85)	Claude Opus 4.6	0.69 (0.57-0.80)	0.04 (-0.09-0.18)	0.547
Claude Sonnet 4	0.73 (0.61-0.85)	Gemini 3 Pro	0.70 (0.59-0.82)	0.03 (-0.09-0.14)	0.676

**Floor-to-ICU within 24 hours: GPT-5 had the highest observed AUC (0.82)**					
**Top observed AUC model**	**Top model AUC (95% CI)**	**Comparator**	**Comparator AUC (95% CI)**	**Delta AUC (95% CI)**	**Paired DeLong P value**
GPT-5	0.82 (0.73-0.91)	GPT-5 mini	0.57 (0.46-0.67)	0.25 (0.13-0.38)	<0.001

GPT-5	0.82 (0.73-0.91)	Claude Sonnet 4	0.61 (0.47-0.75)	0.21 (0.07-0.35)	0.004
GPT-5	0.82 (0.73-0.91)	Claude Sonnet 4.6	0.65 (0.52-0.77)	0.17 (0.04-0.30)	0.009
GPT-5	0.82 (0.73-0.91)	Claude Opus 4.6	0.68 (0.56-0.79)	0.14 (0.03-0.25)	0.009
GPT-5	0.82 (0.73-0.91)	Gemini 3 Pro	0.69 (0.56-0.81)	0.13 (0.00-0.26)	0.051

eTable 5. Estimated single-reviewer chart-screening savings for all models at 80% sensitivity

72-hour return to admission (n = 191; full review time = 15.9 h)				
**Model**	**Threshold**	**Sensitivity**	**Cases Screened Out**	**Time savings**
Claude Sonnet 4	0.75	81.0%	99 (51.8%)	8.2 h
Claude Sonnet 4.6	0.20	90.5%	55 (28.8%)	4.6 h
Claude Opus 4.6	0.15	81.0%	69 (36.1%)	5.8 h
Gemini 3 Pro	0.10	95.2%	40 (20.9%)	3.3 h
GPT-5	0.25	81.0%	77 (40.3%)	6.4 h
GPT-5 mini	0.10	100.0%	4 (2.1%)	0.3 h

Floor-to-ICU within 24 hours (n = 97; full review time = 8.1 h)				
**Model**	**Threshold**	**Sensitivity**	**Cases Screened Out**	**Time savings**
Claude Sonnet 4	0.15	100.0%	0 (0.0%)	0.0 h
Claude Sonnet 4.6	0.10	100.0%	12 (12.4%)	1.0 h
Claude Opus 4.6	0.10	100.0%	30 (30.9%)	2.5 h
Gemini 3 Pro	0.10	83.3%	35 (36.1%)	2.9 h
GPT-5	0.20	94.4%	43 (44.3%)	3.6 h
GPT-5 mini	0.10	94.4%	10 (10.3%)	0.8 h

eFigure 1: Model performance across (A) academic and (B) network strata for ED returns to admission within 72 hours

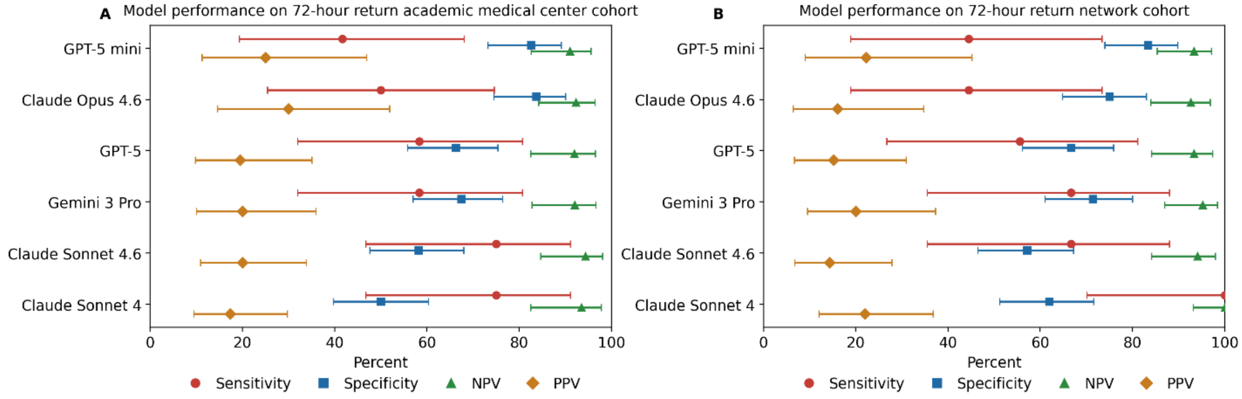

eFigure 2: Pairwise agreement among physician reviewers and large language models across adjudicated emergency care cases. Heatmap cells show Gwet AC1 coefficients for pairwise agreement between individual physician reviewers and each large language model, as well as reviewer-reviewer and model-model comparisons, across the pooled 72-hour return to admission and floor-to-intensive care unit transfer cohorts. Higher values indicate greater agreement. Blank cells indicate reviewer pairs without overlapping reviewed cases.

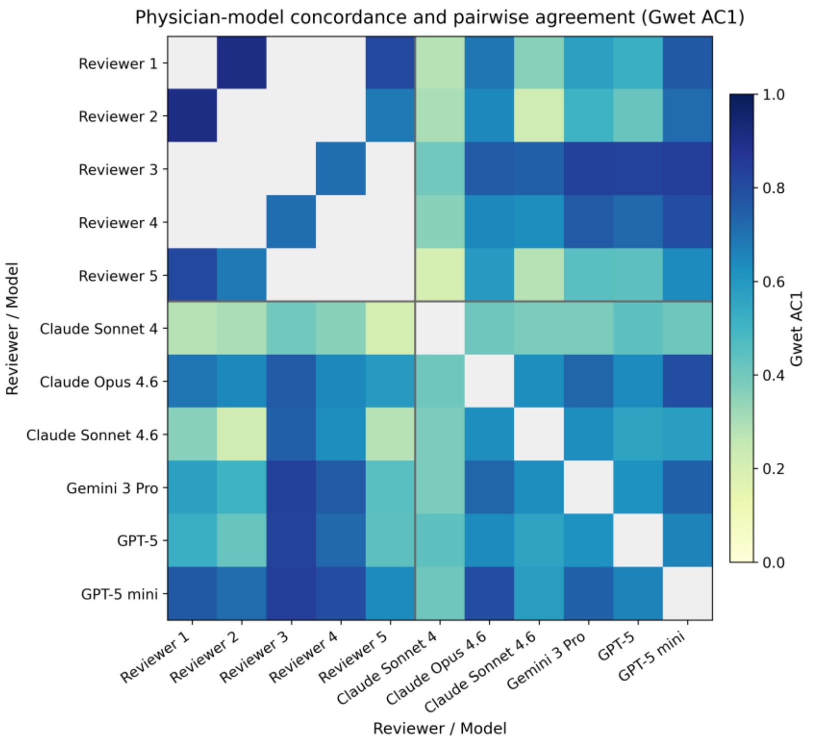

Supplement: Supplement 1. — eMethods. eTable 1. Strata demographics in 72-hour return cohort: Academic medical center vs network eTable 2. Full confusion matrix for all LLMs eTable 3. All model performance findings with 95% confidence intervals eTable 4. Pairwise DeLong comparisons of the highest-observed AUC model versus the remaining models within each cohort eFigure 1. Model performance across academic and network strata for ED returns to admission within 72 hours eTable 5. Estimated single-reviewer chart-screening savings for all models at 80% sensitivity eFigure 2. Pairwise agreement among physician reviewers and large language models across adjudicated emergency care cases [file jamanetwopen-e2620939-s001.pdf]
