# Supplementary material for: Screening for Missed Opportunities for Diagnosis in the ED Using eTriggers and Large Language Models
Source: JAMA Netw Open. 2026 Jun 29;9(6):e2620939. doi: 10.1001/jamanetworkopen.2026.20939 (PMC13316604; doi:10.1001/jamanetworkopen.2026.20939)
Supplement: Supplement 2. — Data Sharing Statement [file jamanetwopen-e2620939-s002.pdf]

Data Sharing Statement

Marks. Screening for Missed Opportunities for Diagnosis in the ED Using eTriggers and Large Language Models. *JAMA Netw Open*. Published June 29, 2026.
doi:10.1001/jamanetworkopen.2026.20939

Data
Data available: No
